# Characterizing Fungal Decay of Beech Wood: Potential for Biotechnological Applications

**DOI:** 10.3390/microorganisms9020247

**Published:** 2021-01-26

**Authors:** Ehsan Bari, Katie Ohno, Nural Yilgor, Adya P. Singh, Jeffrey J. Morrell, Antonio Pizzi, Mohammad Ali Tajick Ghanbary, Javier Ribera

**Affiliations:** 1 Department of Wood Science and Engineering, Section of Wood Microbiology and Genetic, Technical Faculty of No. 1, Mazandaran Branch, Technical and Vocational University (TVU), Sari 4816831168, Iran; 2 USDA Forest Service, Forest Products Laboratory, One Gifford Pinchot Drive, Madison, WI 53726, USA; katie.m.ohno@usda.gov; 3 Department of Forest Products Chemistry and Technology Division, Forest Industry Engineering, Forestry Faculty, Istanbul University Cerrahpaşa, 34473 Istanbul, Turkey; yilgorn@istanbul.edu.tr; 4 Scion, Rotorua 3046, New Zealand; adyasingh@hotmail.com; 5 National Centre for Timber Durability and Design Life, University of the Sunshine Coast, Brisbane 4102, Australia; jmorrell@usc.edu.au; 6 ENSTIB-LERMAB, University of Lorraine, BP 21042, CEDEX 09, 88051 Epinal, France; antonio.pizzi@univ-lorraine.fr; 7 Department of Mycology and Plant Pathology, College of Agronomic Sciences, Sari Agricultural Sciences and Natural Resources University, Sari 4818166996, Iran; m.tajick@sanru.ac.ir; 8 Laboratory for Cellulose & Wood Materials, Empa-Swiss Federal Laboratories for Materials Science and Technology, CH-9014 St. Gallen, Switzerland

**Keywords:** white-rot, brown-rot, soft-rot, biological treatment, lignin degradation, beech

## Abstract

The biotechnological potential of nine decay fungi collected from stored beech logs at a pulp and paper factory yard in Northern Iran was investigated. Beech blocks exposed to the fungi in a laboratory decay test were used to study changes in cell wall chemistry using both wet chemistry and spectroscopic methods. *Pleurotus ostreatus*, *P. pulmonarius*, and *Lentinus sajor-caju* caused greater lignin breakdown compared to other white-rot fungi, which led to a 28% reduction in refining energy. *Trametes* *versicolor* caused the greatest glucan loss, while *P. ostreatus* and *L. sajor-caju* were associated with the lowest losses of this sugar. Fourier transform infrared spectroscopy (FTIR) analyses indicated that white-rot fungi caused greater lignin degradation in the cell walls via the oxidation aromatic rings, confirming the chemical analysis. The rate of cellulose and lignin degradation by the *T.* *versicolor* and *Pleurotus* species was high compared to the other decay fungi analyzed in this study. Based on the above information, we propose that, among the fungi tested, *P. ostreatus* (27.42% lignin loss and 1.58% cellulose loss) and *L. sajor-caju* (29.92% lignin loss and 5.95% cellulose loss) have the greatest potential for biopulping.

## 1. Introduction

Biotechnology is playing an increasingly important role in the field of biomaterials. Recent technical advancements in biomaterials, ranging from evaluation for biomaterial use to address environmental issues, continue to gain interest. The degradation of lignocellulosic biomass is efficiently achieved in nature by many organisms, among which, filamentous fungi are considered key primary degraders [1]. Microorganisms play an increasingly important role in the utilization of lignocellulosic materials via bioremediation [2], biorefining [3,4], bioincising [5,6], bioengineering [7], biofuels [8], and biopulping [9]. These organisms, especially filamentous fungi, have the potential to play key roles in reducing industrial pollution. They also aid in the cost-effective utilization of pulp and paper wastes, especially of medium density fiber boards (MDF) [10,11]. A number of white-rot fungi, especially *Ceriporiopsis subvermispora* [12], *Trametes hirsuta* [13], and *T. versicolor* [14], have shown promise as pretreatment agents for biopulping.

Pretreatment is a crucial step in the conversion of lignocellulosic biomass to fermentable sugars and biofuels. Compared to thermal/chemical pretreatment, fungal pretreatment reduces the difficulty of treatment of lignocellulosic biomass by mean of lignin-degrading microorganisms and, thus, potentially provides an environmentally friendly and energy-efficient pretreatment technology for biofuel production [15]. The potential benefits of fungal pretreatment of wood, especially in biochemical pulping, can be the reduction in pulping time, kappa number, and bleaching chemical consumption, including decreased lignin content of the pulp, reduced consumption of bleaching chemicals [16,17,18], an increase in the strength properties of pulp [19,20], and, also, lower levels of environmental pollution. In fact, biopulping can result in better-quality pulp in terms of fiber flexibility, compressibility, conformability, and collapsibility [21]. During the past four decades, several publications have addressed various aspects of biopulping [22], reporting on the significant reduction of chemicals, manufacturing, and energy costs when using white-rot fungi such as *C. subvermispora*, *Dichomitus squalens*, *Merulius tremellosus*, and *Phlebia brevispora*.

While fungi have great potential for these applications, relatively few species have been explored. Additional fungal species could have potential biotechnological applications. However, it is not feasible to examine all possibilities. One alternative is to take a more targeted approach and examine fungal species that have already shown some ability to compete with other organisms to colonize and grow on a given wood substrate [23]. A prime location to explore possible fungal candidates is in facilities where timber is stored prior to processing.

Certain fungi and bacteria have evolved strategies to degrade wood [24,25,26,27] using a range of enzymes and utilize its cell wall components for their nutrition. Wood cell walls consist of three main types of polymers: cellulose, hemicellulose, and lignin. These polymers are arranged in a way that cellulose and hemicelluloses are protected by the more difficult to cleave lignin and the cell wall becomes compact, with intermolecular pores measuring around 2 nm in size in the processed (Kiln-dried) wood. The pores are too small for much larger fungal enzymes to enter the cell wall. To overcome this barrier, wood-degrading microorganisms deploy small molecular weight substances [28,29], which can penetrate the cell wall and at least partially depolymerize lignin in advance of an enzymatic attack.

Wood-degrading fungi have been classified into three groups: white-rot fungi, brown-rot fungi, and soft-rot fungi. These categories are based primarily on the visual appearance and texture of degraded wood, micromorphological patterns produced during cell wall degradation, and the enzymatic capacity of fungi to degrade wood cell wall polymers, particularly lignin. White and brown-rot fungi belong to basidiomycetes, and soft-rot fungi belong to Ascomycetes and Deuteromycetes. Depending upon the micromorphological patterns of decay produced, white-rot fungi have been placed into two groups: simultaneous degraders and selective (preferential) degraders. Simultaneous degraders remove all cell wall components (cellulose, hemicellulose, and lignin) more or less simultaneously. Consequently, the cell wall is progressively eroded by hyphae present in the cell lumen. Selective degraders preferentially remove lignin. The white-rot fungi causing this type of cell wall degradation have received much attention by workers engaged in fungal biotechnological research [11], and selected fungi have been employed in biopulping in an attempt to reduce the amount of chemicals traditionally used for pulping and, thus, pulping-associated costs, as well to minimize the adverse environmental impacts of pulping chemicals. Brown-rot fungi rapidly and extensively depolymerize cellulose and hemicellulose, leaving behind lignin, which is only partially modified. Due to the extensive depolymerization of cellulose, considerable strength losses can occur in the degraded wood, even at incipient stages of decay. Brown-rot fungi can degrade all cell wall regions, except the lignin-rich middle lamella, which appears to resist degradation. The degraded wood appears dark in color mainly because of the presence of the increased relative proportion of lignin residues. Brown-rotted regions in the degraded wood can readily be spotted when sections are viewed under the polarization mode of the light microscope. Such regions do not display birefringence and appear dark because of the loss of cellulose. The biotechnological potential of these fungi has also been explored; however, their application has been limited compared to selective white rotters [11]. Two micromorphologically different patterns are produced by soft-rot fungi: Type I and Type II. Type I is characterized by the formation of cavities in wood cell walls, and in Type II, hyphae present in the cell lumen cause cell wall erosion, and in this respect, the micromorphological appearance of the eroding cell walls is similar to that produced by simultaneous white rotters. In a Type I attack, characteristic cavities are produced following penetration of hyphae from the cell lumen into the cell wall and their alignment along cellulose microfibrils, a very characteristic feature of this type of soft-rot decay. As cavities are formed in the secondary cell wall and not in the middle lamella, the lignin concentration and type are considered to influence cell wall degradation by Type I soft-rot fungi. In the Type II attack, all cell wall regions are eroded in the advanced stages of decay, except the middle lamella, indicating that lignin also influences this type of soft-rot decay. Type II is common in hardwoods, which differ from softwoods in cell wall lignin concentration and lignin type. The biotechnological potential of soft-rot fungi remains unexplored. The enzymatic and biochemical aspects of wood decay fungi have been reviewed by several workers—more recently, by Daniel [25].

The information generated can serve as a platform for developing biotechnological processes suitable for application in wood biomass industries based on the use of locally available fungi. Hence, this study evaluates eight decay fungi isolated from beech logs stored in a pulp and paper factory yard in Northern Iran. The post-fungal exposure composition of beech wood cell walls was analyzed using biological, chemical, and photochemical analyses. The goal of this study was to assess these decaying fungi in their ability to degrade different wood cell wall components, which could offer a clue as to which of the tested isolates would be best-suited for pulping in relation to biotechnological applications. 

## 2. Materials and Methods

### 2.1. Fungal Isolation

Freshly formed fruiting bodies were collected from stored beech logs in a pulp and paper factory in Northern Iran and maintained on ice until processed. Tentative species identifications were performed following the previously described procedures [30,31]. Molecular identifications were confirmed by removing approximately 20 mg of the interior of each fruiting body and placing it on water agar for 3–5 days at room temperature. Hyphal strands growing from the original fruiting body material were then transferred to 1% malt extract agar (Merck, Darmstadt, Germany) and incubated for 1 week. Approximately 10 mg of fungal growth from the pure culture was transferred to 1% potato dextrose media and grown for 3–5 days. Fungal cultures were identified according to the previously described methods [32,33]. Pure fungal cultures were maintained on 2% malt extract agar in Petri dishes and stored at 4 °C for further testing.

### 2.2. Wood Decay Capabilities

Beech sapwood (*Fagus sylvatica*) lumber was air-dried to 23% ± 2% moisture content (MC) and cut into 54 specimens (30 × 10 × 5 mm) for testing. The wood samples were oven-dried at 103 ± 3 °C and weighed before being sterilized at 121 °C for 20 min and exposed to the test fungi. The fungi were inoculated on malt extract agar in Petri dishes and incubated until the fungus covered the surface. Glass rods were placed on the agar surface, and then, the test blocks were placed on the rods following the procedures of European Standard EN-113 [34], as modified by Bravery [35]. The blocks were incubated for 60 days at 22 ± 2 °C and 65% ± 5% relative humidity (RH). At the end of this period, mycelia were removed from the block surfaces, and the blocks were weighed before being oven-dried at 103 °C and weighed again. The weight after fungal exposure was used to calculate the moisture content to ensure that the blocks were above a 30% moisture content and, therefore, at levels suitable for a fungal attack. Differences between the initial and final oven dry weights were used to calculate the fungal-associated wood weight loss. Each fungus was evaluated on six blocks, along with non-fungal-exposed controls.

### 2.3. Chemical Analysis

Control and fungal-exposed blocks were ground through a 40-mesh screen, and the resulting wood powder was used to determine the acid insoluble lignin content by digestion in 72% sulfuric acid according to TAPPI Method T222 [36], cellulose content of the wood by acid digestion according to TAPPI method T17 [37], and carbohydrate content of the extractive free wood according to TAPPI Standard T249 [38] using a modified method for the carbohydrate analysis [39]. The average chemical composition loss of the decayed wood was calculated as a percent of the corresponding values in the non-exposed samples. The pH of the samples was determined by placing about 1 g of ground wood in 25-mL distilled water and storing overnight at room temperature before measuring the pH with a pH meter. All analyses were performed in triplicate.

### 2.4. Fourier Transform Infrared Spectroscopy (FTIR)

The effect of fungal decay on the wood chemistry was investigated using FTIR spectroscopy. Sound and fungal-exposed samples of each wood species were ground to pass through a 20-mesh screen, then mixed with potassium bromide and pressed into a pellet that was examined using a Shimadzu 8400s FTIR Spectrometer equipped with a deuterated, L-alanine doped triglycine sulfate (DLaTGS) detector. All samples were examined at a spectral resolution of 4 cm^−1^, with 32 scans per sample. Background scans were also carried out using a blank collector. A rubber band method was used for each spectrum, with baseline correction. The band for CO_2_ was removed to produce a suitable baseline correction [40].

## 3. Results and Discussion

### 3.1. Identification of the Isolate

The results of the molecular identification based on the internal transcribed spacer (ITS) regions of the collected fruiting bodies indicated that six isolates were classified as white-rot, one soft-rot, and one brown-rot fungi. Six isolates *Trametes versicolor* (L: Fr.) Pilát, *Pleurotus ostreatus* (Jacq.: Fr.) Kummer, *Lentinus sajor-caju* (Fr.) Singer, *Pleurotus pilmonarius* (Fr.) Quél., *Fomes fomentarius* (L.) Fr., and *Phanerochaete chrysosporium* Burds. were identified as white-rot fungi; one fruiting body *Xylaria longipes* Nitschke. was identified as a soft-rot fungus; and the final fruiting body was identified as a brown-rot fungus *Coniophora puteana* (Schum.: Fr.) P. Karsten.

*T. versicolor* and *P. chrysosporium* have both been extensively studied as potential preferential lignin degraders for use in biopulping [41]. *F. fomentarius* is a common heart-rotter in standing trees and has a worldwide distribution [16]. *P. ostreatus* is globally distributed and a commonly grown commercial mushroom. *P. pulmonarius* and *L. sajor-caju* are sometimes used synonymously, but neither has been explored for the possible modification of wood properties. *X. longipes* is an ascomycete found on hardwoods in temperate regions in the Northern hemisphere and causes soft rot. The final fungal fruiting body was confirmed as *C. puteana*, which is a common brown rotter in a variety of buildings. It is also extensively used as a test decay fungus.

### 3.2. Wood Decay Test and Fungal Metabolism

The wood moisture contents (MC) at the end of the exposure period ranged from 65.3% to 143.9%, well above the levels required for a fungal attack (Table 1). The moisture contents of blocks exposed to *T. versicolor* were the highest measured. This fungus typically forms a dense mycelial film over blocks in decay tests, thereby helping to increase the moisture levels. The lowest moisture contents were found for the brown-rot fungus (*C. puteana*) and one white-rot fungus (*F. fomentarius*).

The mass losses ranged from 12.7% to 31.6%, depending on the fungus. Interestingly, the lowest weight losses were found for the brown-rot *C. puteana*. This species is commonly used as a test fungus in the EN-113 [34] test, where it causes weight losses well over 20%. The lower weight losses illustrate the variations in decay capabilities among different isolates of the same species. *T. versicolor* was associated with the highest weight losses, followed by *P. pulmonarius*, *L. sajor-caju*, and *X. longipes*. The first three fungi cause white-rot decay, which tends to be more aggressive on hardwoods such as beech. The high weight losses associated with *X. longipes* were interesting, since most soft-rot fungi tend to cause much lower weight losses, especially in tests, where conditions are more closely designed for basidiomycetes. The low weight losses associated with *P. chrysosporium* were interesting, since this fungus has long-been studied for biopulping.

### 3.3. Fungal-Associated Changes in the Wood Chemistry

The changes in the chemical composition of beech following 60 days of fungal decay are presented in Table 1. The lignin content decreased in the blocks exposed to all of the test fungi, with the greatest reductions occurring for *P. ostreatus*, *P. pulmonarius*, or *L. sajor-caju.* All three species caused white rot, which would be expected to utilize lignin. The lignocellulosic-degradative capacities for *Pleurotus* species have been explored on oak [42,43,44] and beech wood [45,46,47,48], and it has been suggested that these fungi modify their decay modes (i.e., simultaneous to selective rot), depending on the environmental conditions [45,49,50,51]. The blocks exposed to *T. versicolor*, *F. fomentarius*, and *P. chyrsosporium* also contained lower amounts of lignin than the controls. In previous research [52], the degradation behavior of the white-rot fungus *F. fomentarius* was studied by matrix-assisted laser desorption ionization time of flight (MALDI-TOF) mass spectrometry, and it was demonstrated that this fungus may cause the degradation of walnut wood by soft-rot. White-rot fungi have long been studied for their potential in reducing the costs of pulping; however, complete lignin removal is not often the goal. Rather, the process showing the most promise is the pretreatment of mechanical pulps, where slight reductions in the lignin content have produced significant energy savings. Biopulped chips used to produce mechanical pulps require 25% to 35% less refining energy, 20% less pulping time, and have 20% to 40% improved sheet strength properties [53,54]. Interestingly, blocks exposed to *C. puteana* also contained lower lignin levels, which is inconsistent with the fact that this fungus tends to utilize the carbohydrate fraction, leaving a lignin-enriched residue. Körner et al. [55] showed that the non-sterile incubation of wood chips with *C. puteana* resulted in energy savings of about 40% during the refining of wood chips and a three-fold increase in bending strength of fiber boards, while water absorption and thickness swelling were reduced by more than half. However, galactan and glucan breakdown by *C. puteana* was observed, as is typical for an initial brown-rot attack [23,56].

An analysis of the arabinan, galactan, and xylan residues showed relatively minor changes in their contents compared with mass losses and no consistent trends. The levels should change relatively little in wood attacked by white-rot fungi, since these fungi tend to utilize the various wood fractions at rates that are approximately similar to those for weight loss. The levels of these three sugars would be expected to decline in wood attacked by either the brown or soft-rot fungi, since these species tend to preferentially attack carbohydrates, especially the hemicellulose components.

The glucan residues and total carbohydrates declined in the blocks exposed to all of the fungi, except *P. ostreatus*, although the differences were sometimes slight. The glucan declines were greatest with *T. versicolor*, while the losses by *P. pulmonarius*, *F. fomentarius*, *X. longipes*, and *C. puteana* were similar. The total carbohydrate levels were similar in all blocks at the end of the fungal exposure period, except for those exposed to *T. versicolor*, suggesting that the components were being utilized as they were released from the lignocellulose matrix. 

The pH of the fungal-decayed beech varied from 3.21–4.71 and was much lower than that of the control (5.21). These results are consistent with the tendency of fungi to acidify the surrounding environment as a function of growth as they degrade the polymers to release the monomeric elements.

### 3.4. IR Spectroscopy Analyses of Degraded Wood

**Figure 1:**
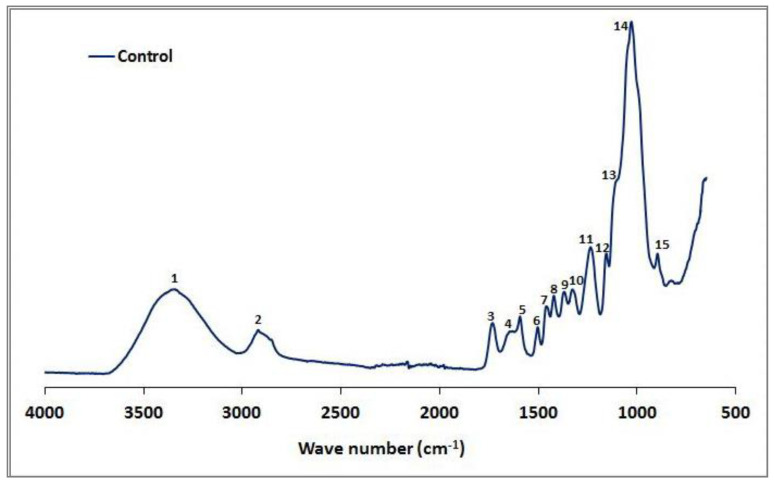
The IR spectra of the control beech wood.

The FTIR spectra of the control beech sample are shown in Figure 1, and potential linkages in the spectra are described (Table 2). However, our results differed from those found in previous studies (e.g., Pandey and Pitman [57]). The high xylan content in hardwoods such as beech produced a strong carbonyl band at 1732 cm^−1^ (3), and the bands at 1318 cm^−1^ (10) and 1233 cm^−1^ (11) reflect the C1-O vibration in syringyl derivatives (Table 3).

**Figure 2:**
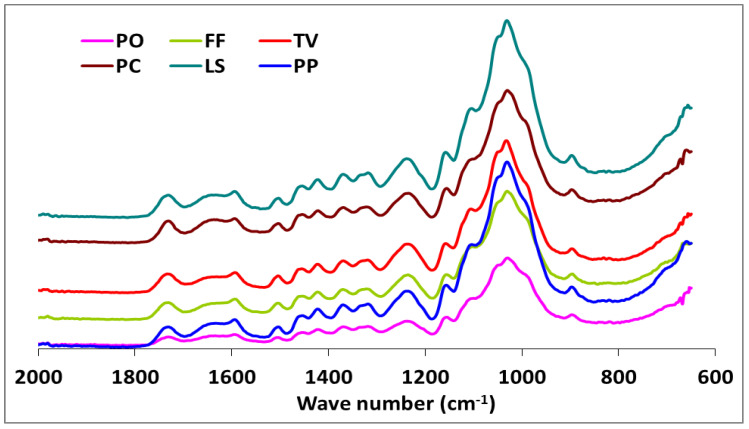
The comparison FTIR spectra of beech wood exposed to Pleurotus ostreatus (PO), Trametes versicolor (TV), Lentinus sajor-caju (LS), Pleurotus pilmonarius (PP), Fomes Fomentarius (FF), and Phanerochaete chrysosporium (PC).

**Figure 3:**
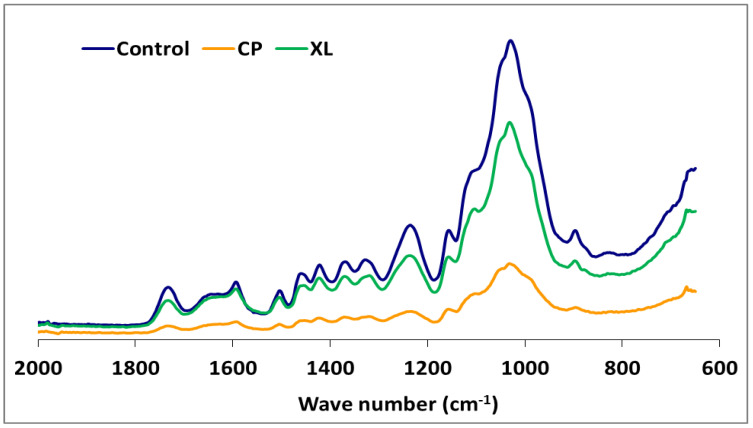
The comparison FTIR spectra of beech wood with or without exposure to *Xylaria longipes* (XL) or *Coniophora puteana* (CP).

The IR spectra of white-, brown-, and soft-rot fungal-decayed beech are shown in Figure 2 and Figure 3. There were no major differences in the spectra of beech wood exposed to the white-rot fungi, which is consistent with the tendency of white-rot fungi to completely utilize decomposition products. However, differences in intensity were noted for the 1592 and 1504 cm^−1^ bands, which represent the aromatic skeletal vibrations (Figure 2). The fungal-decayed beech wood IR spectra corresponded to the relative intensities of aromatic skeletal vibrations against the typical bands for carbohydrates (Table 3). The peak at 1504 cm^−1^ was used as a reference peak for lignin, and the ratio between this peak and the carbohydrate-related peaks at 1732 cm^−1^, 1367 cm^−1^, 1155 cm^−1^, and 895 cm^−1^ was used as a measure of decay [57,58].

Although the highest weight losses were associated with *T. versicolor*, the FTIR analysis indicated that the most substantial carbohydrate losses occurred in specimens attacked by *C. puteana* (Table 2 and Figure 3). Despite the decreases observed in the relative intensities of the aromatic skeletal vibrations against the typical bands for carbohydrates for all fungi tested (Table 3), *C. puteana* was associated with a greater xylan degradation (Table 1). Xylans are characteristic of hardwood hemicelluloses. Small increases determined in the relative intensities of the ratios in 1504/1367 and 1504/1155 were 3.64% and 7.08%, respectively. The two bands at 1367 and 1155 cm^−1^ indicated the presence of hemicellulose and cellulose (Table 2). However, the small increase in the relative intensity of the ratio at 1504/895 cm^−1^ indicated that C-H deformation in cellulose was not as important as suggested by the traditional chemical analysis (Table 3). The small increase in the 1504/895 ratio indicated that *C. puteana* degraded cellulose more slowly than the hemicellulose polyoses. Brown-rot decay is generally presumed to rapidly depolymerize polysaccharides, while lignin remains as a complex and modified polymer [59,60]. However, Kim et al. [61] found that *C. puteana* degraded hardwood cell walls, including the middle lamellae, which is mainly composed of lignin. Lignin was the most resistant polymer to biodegradation in beech samples exposed to *C. puteana*; however, a chemical analysis suggested some lignin modification (Table 1). These changes were consistent with the results obtained in recent studies suggesting that brown-rot fungi have much greater effects on lignin structures. Hemicellulose modification by brown-rot fungi varies with wood species [59]. The ratio of 1504/1732 was 1.137 for the control specimens and declined to 0.943, 0.973, 0.933, 1.000, 0.987, and 0.922 for the species exposed to *T. versicolor, P. ostreatus, L. sajor-caju, F. fomentarius, P. pulmonarius*, and *P. chrysosporium*, respectively (Table 3). 

These results were consistent with the ability of white-rot fungi to degrade lignin and modify the aromatic units [57,62,63]. Faix et al. [62] noted decreasing intensities of the bands represented by aromatic skeletal vibrations, indicating structural changes and loss of these units during white-rot degradation. However, the ratios of 1504/1367, 1504/1155, and 1504/895 were higher in blocks exposed to *T. versicolor*, *L. sajor-caju*, and *F. fomentarius* (Table 3). The bands at 1367 and 1155 cm^−1^ represent the presence of hemicellulose and cellulose, while the band at 895 cm^−1^ represents the presence of cellulose (Table 3). Thus, decreased intensities in the ratio of 1504/1732 and increases in the 1504/1367, 1504/1155, and 1504/895 ratios indicated a nonselective degradation by the three white-rot fungi. The ratio of 1504/895 suggested that *T. versicolor* caused the greatest reductions in cellulose, which was consistent with previous reports that this fungus causes a simultaneous white-rot rather than a selective delignification [62,63]. Conversely, the ratios of 1504/1367, 1504/1155, and 1504/895 in the specimens exposed to *P. ostreatus*, *P. pilmonarious*, and *P. chrysosporium* decreased compared to the control, suggesting preferential lignin degradation (Table 1).

White-rot fungi secrete both oxidative and hydrolytic extracellular lignocellulolytic enzymes that are responsible for the decomposition of both lignin and carbohydrates. The possibility of a complementary action between these enzymes is directly interrelated to the structural configuration of the plant cell walls [68]. However, research has shown that lignin is oxidized and degraded by an oxidative ligninolytic system [69]. Whereas some white-rot fungi decompose lignin to a greater extent than cellulose [63], significant cellulose degradation can occur by soft-rot fungi [70]. Our results suggested that *T. versicolor*, *L. sajor-caju*, and *F. fomentarius* are simultaneous degraders on this substrate, with the degradation rate of lignin and cellulose being similar for *T. versicolor*. The alteration of the ratios for 1504/1732 were (−) 17.06, (−) 17.94, and (−) 13.19 in the specimens exposed to *T. versicolor, L. sajor-caju*, *and F. fomentarius*, respectively. However, the ratios for 1504/895 were (+) 11.74, (+) 6.08, and (+) 2.73 in the same specimens (Table 3). As seen in the Table 1 and Figure 3, lignin degradation was greater than carbohydrate degradation, which concurs with the suggestion of Schwarze [71] that white-rot fungi access carbohydrates by degrading lignin. 

The FTIR spectra for the soft-rot fungus *Xylaria longipes* were similar to those for *T. versicolor*, except that the soft-rot fungus appeared to cause more cellulose and less lignin decomposition (Table 3). Blanchette [70] suggested that soft-rot fungi do not completely degrade the lignin in the middle lamella, although they are capable of modifying it. A type 1 soft-rot attack clearly affects the lignin integrity, and this effect should be fairly prominent in blocks with this level of mass loss.

## 4. Conclusions

The information gained in this research work has potential for pulping and related biotechnological applications, primarily for those fungi that remove lignin in preference to cellulose. During the pulping process, chemicals have traditionally been used to remove lignin from wood chips, which has damaging effects on our environment. This concern has led to attempts at biopulping using particularly selective white-rot fungi, which preferentially degrade lignin. In addition to protecting the environment, biopulping can generate economic benefits and, also, improve the pulp and paper quality [72]. Therefore, we consider our work to be of significant value in technological developments aimed at improving and innovating pulp-processing methods and recommend the following fungi for biopulping in the order listed: *P. ostreatus* and *L. sajor-caju*. The reason for this ranking is that, after two months of incubation, the lignin loss was extensive and comparable for the two fungi, with *L. sajor-caju* causing slightly greater lignin loss compared to *P. ostreatus*, whereas *P. ostreatus* caused a much lower loss in cellulose (1.58%) than *L. sajor-caju* (5.95%).

## Figures and Tables

**Table 1:** Mass loss, wood moisture levels, and chemical contents of beech blocks exposed to selected decay fungi in an EN-113 [34] agar block decay test.

	Content (%)								
Category	Control	*Trametes versicolor*	*Pleurotus ostreatus*	*Lentinus sajor-caju*	*Pleurotus pilmonarius*	*Fomes fomentarius*	*Phanerochaete chrysosporium*	*Xylaria longipes*	*Coniophora puteana*
ML	-	31.6	26.3	27.0	25.6	17.4	11.2	26.3	12.7
Lignin	24.10	21.93	17.48	16.89	17.63	21.63	20.28	21.69	20.06
AISL	1.87	2.16	2.02	2.20	2.00	1.94	2.14	2.16	1.91
Araban	1.08	1.03	0.93	0.84	0.85	0.96	1.03	1.04	0.93
Galactan	3.98	2.89	3.21	2.12	2.38	3.31	3.56	3.35	3.58
Glucan	53.7	42.57	52.85	50.50	47.80	46.36	48.41	45.73	49.16
Xylan	11.45	13.45	12.19	12.21	12.15	11.83	11.84	13.33	10.88
Total Carb	64.78	59.94	69.18	65.66	63.18	62.47	64.84	63.46	64.56
Ash	0.03	0.09	0.16	0.02	0.13	0.90	0.03	0.21	0.20
pH	5.21	4.11	3.79	4.71	3.91	4.44	4.88	3.21	3.25
MC	-	143.9	98.6	97.2	93.7	74.8	62.2	98.6	65.3

ML: mass loss, AISL: acid insoluble lignin, and MC: moisture content of the wood.

**Table 2:** Assignments of IR peaks to various cell wall polymer components.

Wave Number (cm^−1^)	Band Assignment	References
3332 (1)	O-H stretching of bonded hydroxyl groups	[2,4,5]
2896 (2)	Symmetric CH stretching in aromatic methoxyl groups and in methyl and methylene groups of side chains	[4,5]
1732 (3)	C=O stretching in xylans (unconjugated)	[1,2,4,5]
1635 (4)	H-O-H deformation vibration of absorbed water and C=O stretching in lignin	[1,5]
1592 (5)	C=C stretching of the aromatic ring (S)Aromatic skeletal vibrations + C=O stretchingS≥ G	[1,4,5]
1504 (6)	C=C stretching of the aromatic ring (G)Aromatic skeletal vibrations in lignin	[1,4,5]
1452 (7)	CH2 deformation vibrations in lignin and xylans	[2,4,5]
1421 (8)	C–H asymmetric deformation in –OCH3Aromatic skeletal vibrations combined with C-Hin plane deformation + C-H deformation in lignin and carbohydrates	[1,3,4]
1367 (9)	C-H deformation in cellulose and hemicelluloses	[3,4,5]
1318 (10)	C-H vibration in cellulose + C1-O vibration insyringyl derivatives	[4,5]
1233 (11)	Acetyl and carboxyl vibrations in xylans and C=O stretching vibrations in lignin	[2,4,5]
1155 (12)	C-O-C vibration in cellulose and hemicelluloses	[2,4,5]
1097 (13)	Aromatic C–H in-plane deformation (typical for S units) and C=O stretch O-H association band in cellulose and hemicelluloses	[2,4,5]
1029 (14)	C=O stretching vibration in cellulose, hemicelluloses, and lignin	[2,4,5]
895 (15)	C-H deformation in cellulose	[3,4,5]

(1) Pandey and Pitman [57], (2) Harrington et al. [64], (3) Faix [65], (4) Schwanninger et al. [66], and (5) Naumann et al. [67].

**Table 3:** Effect of fungal exposure on the FTIR spectra, as shown by ratios between lignin and the various carbohydrate fractions.

	Peak Ratios (%) ^a^			
Fungus	1504/1732	1504/1367	1504/1155	1504/895
*T. versicolor*	86.6	98.3	103.1	111.7
*P. ostreatus*	85.6	94.7	96.9	92.7
*L. sajor-caju*	82.1	103.2	103.8	106.1
*P. pulmonarius*	87.9	93.8	97.3	99.8
*F. fomentarius*	86.1	102.0	103.5	102.7
*P. chrysosporium*	81.1	90.2	91.9	95.2
*C. puteana*	60.3	103.6	107.1	100.6
*X. longipes*	95.4	101.7	106.5	113.6

^a^ The ratios of relative intensities of the aromatic skeletal vibrations against those for the various carbohydrate fractions for the non-fungal-exposed control were 1.14, 0.66, 0.48, and 048 for the ratios of the peak at 1504 cm^−^^1^ with those at 1732, 1367, 1155, and 895 cm^−^^1^.

## Data Availability

All data generated or analyzed during this study are included in this published article. There are no Supplementary Information files.
